# Quantitative analysis of the splice variants expressed by the major hepatitis B virus genotypes

**DOI:** 10.1099/mgen.0.000492

**Published:** 2021-01-13

**Authors:** Chun Shen Lim, Vitina Sozzi, Margaret Littlejohn, Lilly K.W. Yuen, Nadia Warner, Brigid Betz-Stablein, Fabio Luciani, Peter A. Revill, Chris M. Brown

**Affiliations:** ^1^​ Department of Biochemistry, School of Biomedical Sciences, University of Otago, Dunedin, New Zealand; ^2^​ Victorian Infectious Diseases Reference Laboratory, Royal Melbourne Hospital at the Peter Doherty Institute for Infection and Immunity, Melbourne, Victoria, Australia; ^3^​ Systems Medicine, School of Medical Sciences, Faculty of Medicine, University of New South Wales, Sydney, New South Wales, Australia; ^4^​ Department of Microbiology and Immunology, University of Melbourne, Melbourne, Victoria, Australia; ^‡^​ Present address: Dermatology Research Centre, Diamantina Institute, University of Queensland, Brisbane, Queensland, Australia

**Keywords:** HBV, pgRNA, shotgun sequencing, transcriptome assembly

## Abstract

Hepatitis B virus (HBV) is a major human pathogen that causes liver diseases. The main HBV RNAs are unspliced transcripts that encode the key viral proteins. Recent studies have shown that some of the HBV spliced transcript isoforms are predictive of liver cancer, yet the roles of these spliced transcripts remain elusive. Furthermore, there are nine major HBV genotypes common in different regions of the world, these genotypes may express different spliced transcript isoforms. To systematically study the HBV splice variants, we transfected human hepatoma cells, Huh7, with four HBV genotypes (A2, B2, C2 and D3), followed by deep RNA-sequencing. We found that 13–28 % of HBV RNAs were splice variants, which were reproducibly detected across independent biological replicates. These comprised 6 novel and 10 previously identified splice variants. In particular, a novel, singly spliced transcript was detected in genotypes A2 and D3 at high levels. The biological relevance of these splice variants was supported by their identification in HBV-positive liver biopsy and serum samples, and in HBV-infected primary human hepatocytes. Interestingly the levels of HBV splice variants varied across the genotypes, but the spliced pregenomic RNA SP1 and SP9 were the two most abundant splice variants. Counterintuitively, these singly spliced SP1 and SP9 variants had a suboptimal 5′ splice site, supporting the idea that splicing of HBV RNAs is tightly controlled by the viral post-transcriptional regulatory RNA element.

## Data Summary

The raw RNA-sequencing (RNA-seq) libraries for this study have been deposited in the Gene Expression Omnibus (GSE155983). The PacBio circular consensus sequencing reads analysed in this study have been previously published and can be found in the European Nucleotide Archive (PRJEB12450) [1]. The RNA-seq libraries of hepatitis B virus (HBV)-positive biopsy samples of human liver tumours and tissues, and portal vein tumour thrombosis (129, 182 and 92 libraries, respectively), and HBV-infected primary human hepatocytes (83 libraries) and human cultured cells HepaRG (4 libraries) and HepG2-NTCP (11 libraries) were downloaded from the Sequence Read Archive [2–18] (see the metadata in Table S1, available with the online version of this article).

**Impact Statement**

Hepatitis B virus (HBV) infection affects over 257 million people worldwide. HBV is a major cause of liver diseases, including cancer, and there is no cure. Although not critical for HBV replication, some HBV RNAs are spliced and the abundant splice variants have been found previously to be associated with liver cancer. The role of these HBV splice variants is still poorly understood. HBV exists as nine genotypes worldwide with marked differences in replicative capacity and disease sequelae. Whether HBV splice variants vary for the different genotypes is yet to be investigated in depth. Here, we sequenced RNAs from four major HBV genotypes using a cell culture system. We found 6 new and 10 previously known splice variants across these genotypes. Some novel HBV splice variants were present at high levels not only in our cell culture system, but also in HBV-positive liver biopsy samples and HBV-infected primary human cells, suggesting they could be functionally important.

## Introduction

Hepatitis B virus (HBV) is a common human pathogen that is a major cause of liver cirrhosis and liver cancer. The genomic DNA of HBV is approximately 3.2 kb, but can be transcribed into the greater than genome length pregenomic RNA (pgRNA), preC RNA (pcRNA) and long X RNA (lxRNA) [19, 20]. The pgRNA encodes the core (C) and polymerase (P) proteins, whereas the pcRNA encodes the pre-core (PC) protein that is subsequently processed into the hepatitis B e antigen (HBeAg). The HBV genome is also transcribed into several subgenomic transcripts, namely the preS1, preS2, S and X mRNAs. The preS1, S2 and S mRNAs encode the three surface (S) structural proteins of the HBV particles and subviral particles (HBsAg). The X mRNA encodes the HBx protein.

Many strains of HBV have arisen from distinct geographical distributions of the world. This is partly due to the long history of virus–host coevolution (over 50 000 years) and the lack of proofreading function of the viral reverse transcriptase [21–23]. These strains were grouped into nine major genotypes (A to I) and putative J, and about 30 sub-genotypes [23, 24]. There are marked differences in replication phenotype and disease natural history across HBV genotypes [25, 26], yet the pathogenicity of different HBV genotypes and their implications for treatment are still not fully understood. For example, it is possible that severe liver injury caused by genotype C is related to its high replication capacity [27] and/or its more frequent mutations at the basal core promoter (BCP) and pre-core regions [28, 29].

In addition, different HBV genotypes may produce distinct spliced transcript isoforms whose precise roles are largely unknown [30–33]. At least 18 spliced transcripts of pgRNA [1, 33–44] and 4 spliced transcripts of preS2/S [45, 46] were identified in various sources including liver, serum and transfected cells. Interestingly, a recent study showed that HBV RNA splicing is more efficient in human hepatoma cells than other tested cell types [33]. Furthermore, spliced pgRNA SP1 is the most commonly detected [35, 36, 38, 40, 47–50], although SP3 and SP9 have also been commonly observed in some studies [1, 32, 33]. These abundant HBV splice variants were previously shown to produce duplex linear DNA and apparent ssDNA species, but rarely relaxed circular DNA [41 ]. However, their roles in the normal viral life cycle are still unclear.

Notably, HBV splice variants can be encapsidated to form defective viral particles, with replication and envelopment requiring polymerase and envelope proteins supplied *in trans* by wild-type HBV [37, 39, 40, 51]. The SP1 transcript also encodes the hepatitis B spliced protein (HBSP) [47], as well as a truncated (by one amino acid) PC p22 protein that has been shown to inhibit wild-type HBV replication by interfering with wild-type capsid assembly [52]. The HBSP is a fusion product of the first 46 amino acid residues of the P protein and 47 amino acid residues from a distinct reading frame. A recent breakthrough study showed that HBSP could reduce liver inflammation *in vivo* [50]. Three other splice variants that have coding potential are SP7, SP10 and SP13. SP7 encodes the hepatitis B doubly spliced protein (HBDSP), a putative pleiotropic activator, which has been shown to increase replication of wild-type HBV in co-transfection cell culture experiments [53]. SP13 encodes the polymerase-surface fusion protein (P-S FP), a structural protein that could substitute the large HBV surface protein [54]. This fusion protein could inhibit HBV replication and may play a role in persistent infection. Interestingly, SP10 could also act as a functional RNA that reduces wild-type HBV replication through interaction with the TATA box binding protein [55].

An increasing number of studies have shown that the HBV splice variants are associated with the development and recurrence of hepatocellular carcinoma (HCC) [49, 56, 57], and poor response to interferon treatment [43]. Therefore, we aimed to utilize RNA-sequencing (RNA-seq) on cells that had been transfected with replication-competent clones of different HBV genotypes to (i) quantify the composition of splice variants at the RNA level, (ii) investigate the effects of sequence variations on splicing efficiency, (iii) determine the usage of splice sites, and (iv) understand the host response to viral replication across the major HBV genotypes A to D.

## Methods

### Cell culture

Cell culture and transfection experiments were carried out as previously described with the following modifications [25]. Huh7 cells were seeded in six-well plates at partial confluence. After overnight incubation, the cells were transiently transfected with pUC57 constructs harbouring 1.3-mer HBV genomes (genotypes A2, B2, C2 and D3) using FuGENE 6 transfection reagent, according to the manufacturer’s instructions (Promega). The generation of plasmids has been previously described and this transient expression system relies on the endogenous promoters of HBV for transcription [25]. The empty pUC57 vector was used as a control. Two independent biological replicates were performed, which included two technical replicates for each treatment.

### RNA-seq

**Fig. 1.**
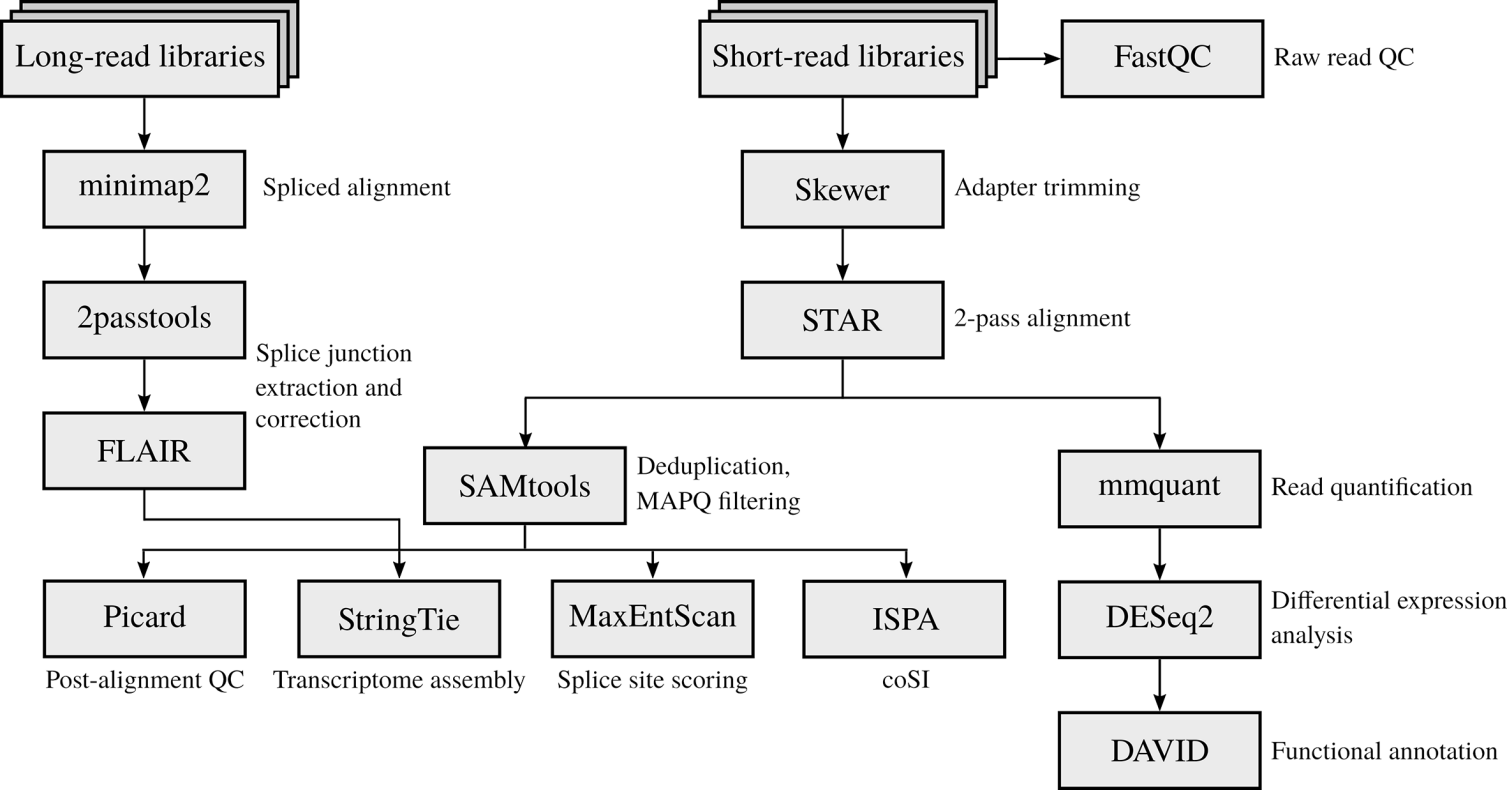
RNA-seq analysis of the HBV and host transcriptomes. QC checking of the paired-end RNA-seq libraries was carried out using fastqc. Adapter sequences were trimmed from the RNA-seq reads using skewer. Trimmed reads were aligned to the human genome and HBV pgRNAs using star in 2-pass mode. Duplicated and multi-mapped reads were discarded from the binary alignment map (BAM) files using samtools. A post-alignment QC check was performed using picard tools. PacBio CCS reads were aligned to the HBV pgRNA using minimap2. HBV splice junctions were extracted and corrected using 2passtools and flair, respectively. Reference-based transcriptome assembly and quantification were carried out using stringtie, with a post-processing step focusing on the HBV spliced transcript isoforms. Splice site sequence contexts were scored using maxentscan. Completeness of RNA splicing was evaluated using ipsa. Reads mapped to human genes were quantified using mmquant, followed by differential gene expression analysis using deseq2. A list of differentially expressed genes was submitted to the david webserver for functional annotation analysis.

Total RNA samples were purified using an RNeasy kit (Qiagen) and submitted to the Otago Genomics and Bioinformatics Facility at the University of Otago (Dunedin, New Zealand) under contract for library construction and sequencing. The libraries were prepared using a TruSeq stranded total RNA sample preparation kit with Ribo-Zero (Illumina) according to the manufacturer’s protocol, and sequenced using HiSeq 2500 (Illumina), generating 125 bp paired-end reads (see the RNA-seq analysis workflow in Fig. 1).

### Quality control (QC) of RNA-seq

The fastq files were examined using fastqc v0.11.5 [58]. Most files passed most of the analysis modules except ‘per base sequence content’, ‘sequence duplication levels’ and ‘k-mer content’, which are common warnings for Illumina TruSeq reads (fastqc documentation). However, some fastq files failed at per base sequence quality and per base N content due to decrease of the quality score over position 100. Some fastq files also failed at per tile sequence quality due to loss of quality at random positions and cycles, which is likely due to the overloading of the flow cell. Both of these issues should have minimum impact on downstream analysis, because the regions of poor base calling were soft-clipped during alignment. In addition, only uniquely mapped reads were used for gene counting and transcript assembly.

As a post-alignment QC, the mapping statistics of the non-redundant RNA-seq reads were examined. About 60 % of the reads were uniquely mapped reads to the human genome (Table S2). The distribution of aligned reads was then analysed using the CollectRnaSeqMetrics program of picard 2.10.2 (http://broadinstitute.github.io/picard). Over 55 and 27 % of the bases of these reads were mapped to the coding sequences (CDS) and untranslated regions (UTRs), respectively (Table S3). Only 10 % or lower of the bases of the sequencing reads were aligned to intronic or intergenic regions. These metrics are comparable with previous findings [59], indicating that our RNA-seq libraries are reliable.

### Sequence alignment

Adapter sequences were trimmed from RNA-seq reads using skewer v0.2.2 [60]. To detect novel splice junctions, RNA-seq reads were aligned to the human genome and HBV pgRNAs using star v2.7.6a in 2-pass mode [61]. Duplicated reads were removed and uniquely mapped reads were retained using samtools v1.2 [62] or picard MarkDuplicates.

The PacBio circular consensus sequencing (CCS) reads of the whole-genome sequencing of HBV were downloaded from the European Nucleotide Archive (PRJEB12450). This dataset was previously generated from the liver explant and post-transplant blood specimens of a patient with chronic HBV infection in a longitudinal study [1]. The CCS reads were aligned to a pgRNA sequence (HBV genotype D, GenBank accession no. X02496.1) using minimap v2.17 in splice mode [63]. Splice junctions were extracted and corrected using 2passtools and flair, respectively [64, 65]. The BED output file was converted into genotype-specific GTF annotation files using UCSC kentutils [66].

### HBV genotyping

HBV genomic sequence alignment was downloaded from HBVdb [67]. The genomic sequences were converted into pgRNA sequences, split by genotype (A to H), and realigned using muscle v3.7 [68]. A profile hidden Markov model (HMM) was built for each genotype using the hmmbuild program of hmmer v3.3.1 [69]. The RNA-seq reads mapped to HBV were searched against the profile HMMs using nhmmer [70]. A median bit score was calculated for hits to each genotype, in which the highest-scoring genotype was assigned to the RNA-seq BioSamples. As validation, this method accurately predicted the HBV genotypes of the transfected Huh7 and infected primary human hepatocyte (PHH) samples.

### Transcriptome assembly

HBV splice variants were detected using stringtie v1.3.3b [71] and guided by the HBV transcript annotation obtained from the above long-read analysis. Only the splice variants supported by a minimum splice junction coverage of two and with complete, exact match intron chains across independent biological replicates were reported.

Annotations of the spliced transcript isoforms were merged by biological replicates using gtfmerge (https://github.com/Kingsford-Group/rnaseqtools) and gffcompare [72]. Only the assembled spliced transcripts that were found in both biological replicates were reported (intersection of complete, exact match intron chain). After merging the BAM (binary alignment map) files by biological replicates using samtools, a spliced graph of HBV transcripts was plotted using gviz v1.32.0 and genomicfeatures v1.40.1 [73, 74]. Transcription start sites were annotated according to a published cap analysis of gene expression [75].

### Splice site analysis

Splice site sequence contexts were scored using maxentscan [76]. This tool is a key plugin of the Ensembl Variant Effect Predictor [77] and performed the best in a recent benchmark [78]. Splice site mapping frequencies were parsed from the SJ.out.tab file from star. ipsa was used to calculate the completed splicing index (coSI) score of 5′ and 3′ splice sites (https://github.com/pervouchine/ipsa) [79 ]. weblogo 3.5.0 was used to plot the nucleotide frequencies surrounding the splice sites [80].

### Differential gene expression analysis

To examine the reproducibility of the biological replicates, the uniquely mapped reads were first counted and summarized at the gene level using mmquant v1.3 [81]. The correlation of samples was analysed. The Spearman’s correlations between the biological replicates were >0.9, suggesting a good reproducibility (Fig. S1). However, the Spearman’s correlations between biological replicates were smaller than those within the same batch (e.g. A2_rep1 versus A2_rep2 is 0.938, whereas A2_rep1 versus B2_rep1 is 0.996). These results suggest the presence of batch effects, which is likely due to the second biological replicate being performed a year after. This was further examined using principal component analysis (PCA). Indeed, the samples were clustered by batches (Fig. S2).

To resolve the issue of batch effects, read counts were transformed using the vst (variance-stabilizing transformation) function of deseq2 [82]. Transformed read counts were examined using the plotPCA function of deseq2 before and after correction using the removeBatchEffect function of limma [83]. To take batch effects into account, differential-expression analysis was carried out using batch as a linear term in the DESeqDataSetFromMatrix function. Differentially expressed genes were examined using david functional annotation tools v6.8 [84, 85].

### Statistical analysis

Welch two-sample *t*-tests and permutation tests were performed using the exactranktests R package [86, 87]. Plotting was carried out using ggplot2, unless otherwise stated [88].

### Code and data availability

Scripts and data for the analysis can be found at https://github.com/lcscs12345/HBV_splicing_paper_2020.

## Results and Discussion

### Six of sixteen HBV splice variants detected were novel transcripts

**Fig. 2.**
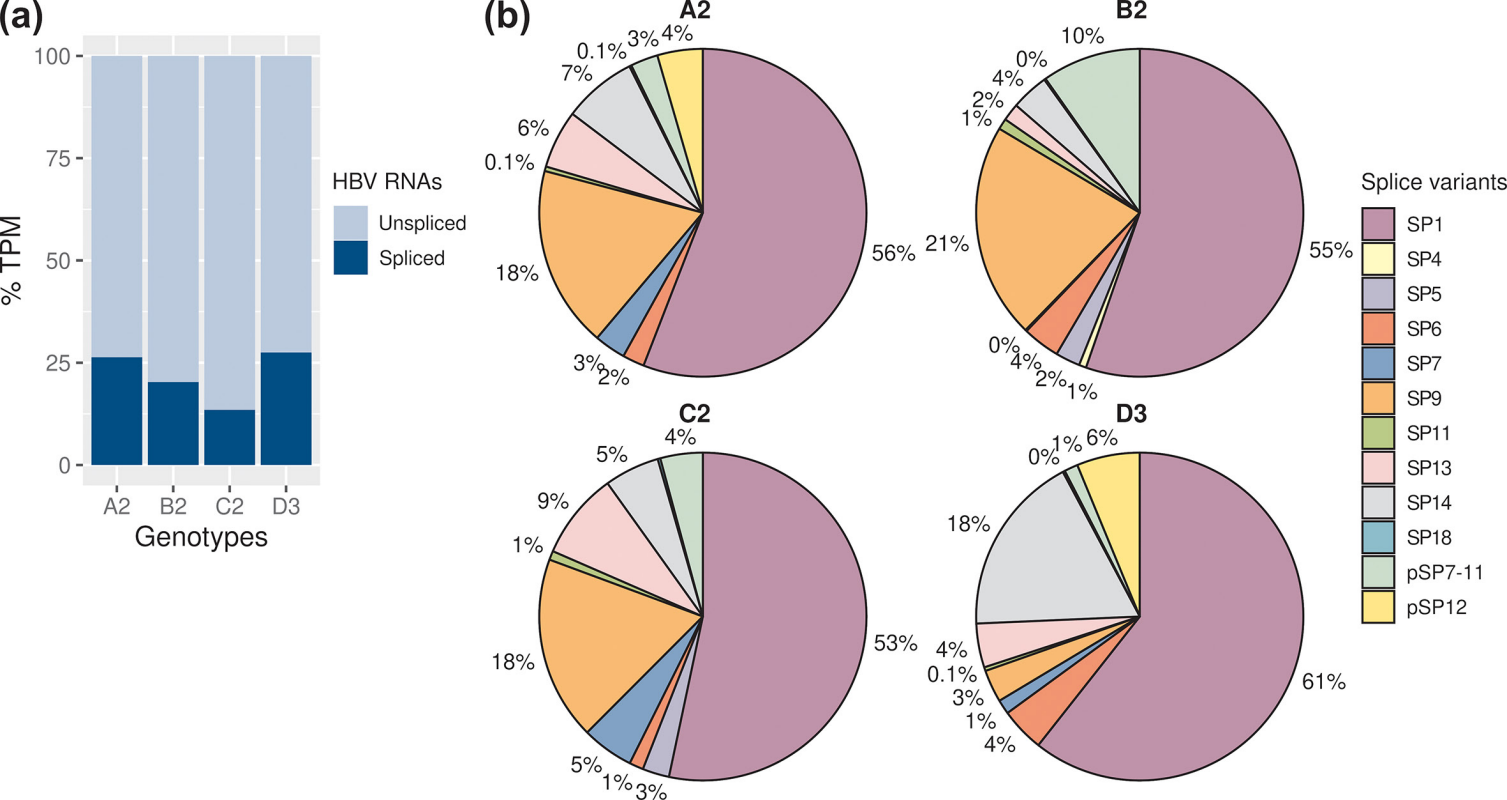
HBV genotypes expressed a wide variety of spliced transcript isoforms. (a) Proportions of the spliced transcripts in HBV RNAs. Only the spliced transcripts present in both biological replicates are shown. (b) Relative abundance of the HBV splice variants in genotypes A to D. See also Tables S4 and S5.

Cells were transfected with four different genotypes and total RNA extracted after 24 h, depleted of rRNAs and deep-sequenced. In addition to the well-established subgenomic and spliced transcripts, this method allowed us to detect spliced transcripts with greater sensitivity. The Huh7 cell transfection system showed that HBV genotypes A to D expressed a large proportion of spliced transcript isoforms. These splice variants represented 13–28 % of the HBV transcriptomes detected (Fig. 2a), showing that HBV splicing was common, and found across the genotypes. HBV genotype B2 expressed the highest level of HBV transcripts, followed by A2, C2 and D3 [4812, 5442, 4708 and 3972 TPM (transcripts per kilobase million mapped reads), respectively; see also Fig. S3, Table S4 for read counts].

**Fig. 3.**
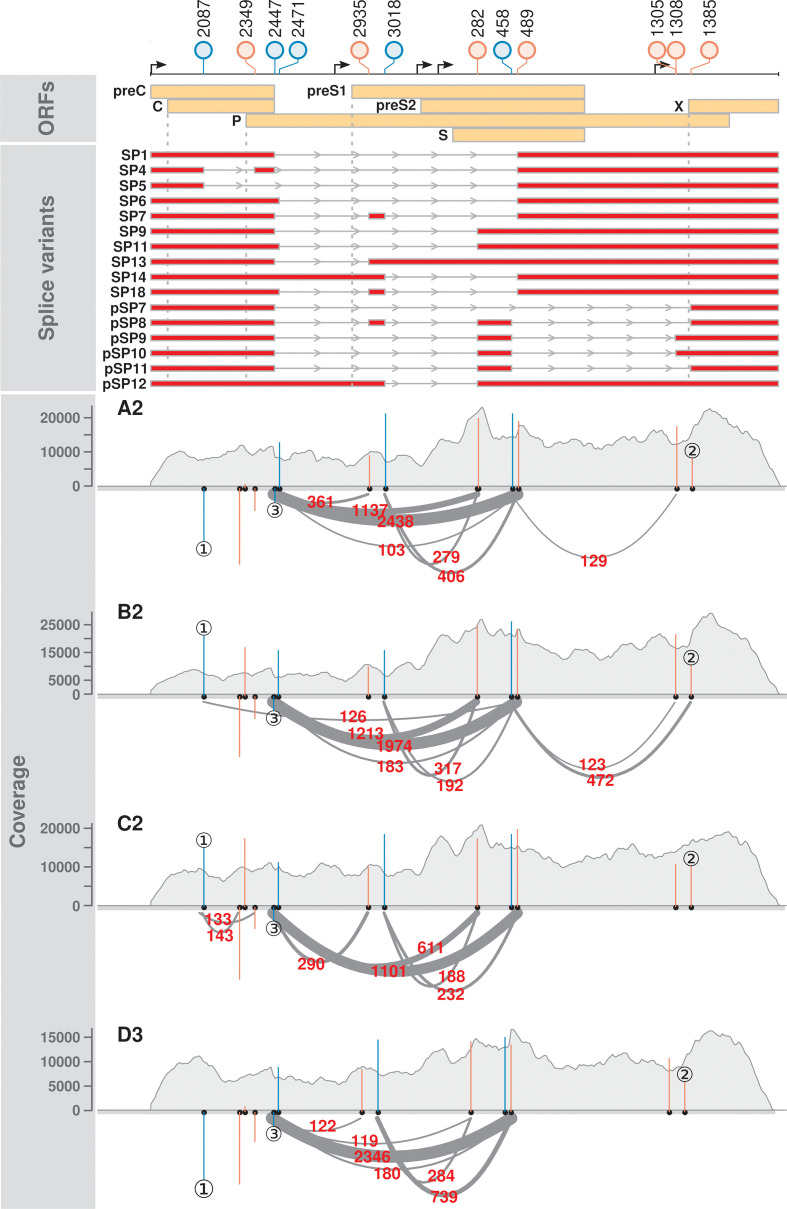
Distinct splicing profiles were observed across the HBV genotypes. The lollipop plot indicates the positions of splice sites relative to the *Eco*RI site of genotype C2. Blue and red colours indicate 5′ and 3′ splice sites, respectively. SP and pSP denote the known and putative spliced pgRNA transcripts, respectively (splice variants panel). These splice variants were reproducibly detected across the independent biological replicates of HBV-transfected Huh7. Grey dotted lines denote the positions of initiation codons of C, P, preS1 and X reading frames (ORFs panel). Read coverage is shown in grey (coverage panel). Arcs represent RNA-seq reads mapped across the splice junctions (supporting read counts in red colour). Only the splice junctions supported by ≥100 reads are shown for readability purposes. Blue and red vertical lines indicate the MaxEntScan scores of the 5′ and 3′ splice sites, respectively (coverage panel). A positive MaxEntScan score predicts a good splice site sequence context, whereas a negative score predicts a poor splice site sequence context. Three main scenarios were observed. ① The presence and absence of spliced reads at position 2087 were predicted by MaxEntScan scores, in which reads were found to map across the 5′ splice sites with strong positive scores (B2 and C2), but not those with strong negative scores (A2 and D3). ② Varying spliced read counts could not be explained by similar scores. ③ Most spliced reads were mapped across a weak splice donor site. See also Fig. S4, Table S8.

A total of 16 splice variants were consistently detected across two independent biological replicates, in which 6 of them were novel (Figs 2b, 3 and S4, labelled pSP). In particular, a novel, singly spliced RNA (pSP12) was expressed at high levels in the genotypes A2 and D3 (4.5–6.2 % of HBV splice variants).

**Fig. 4.**
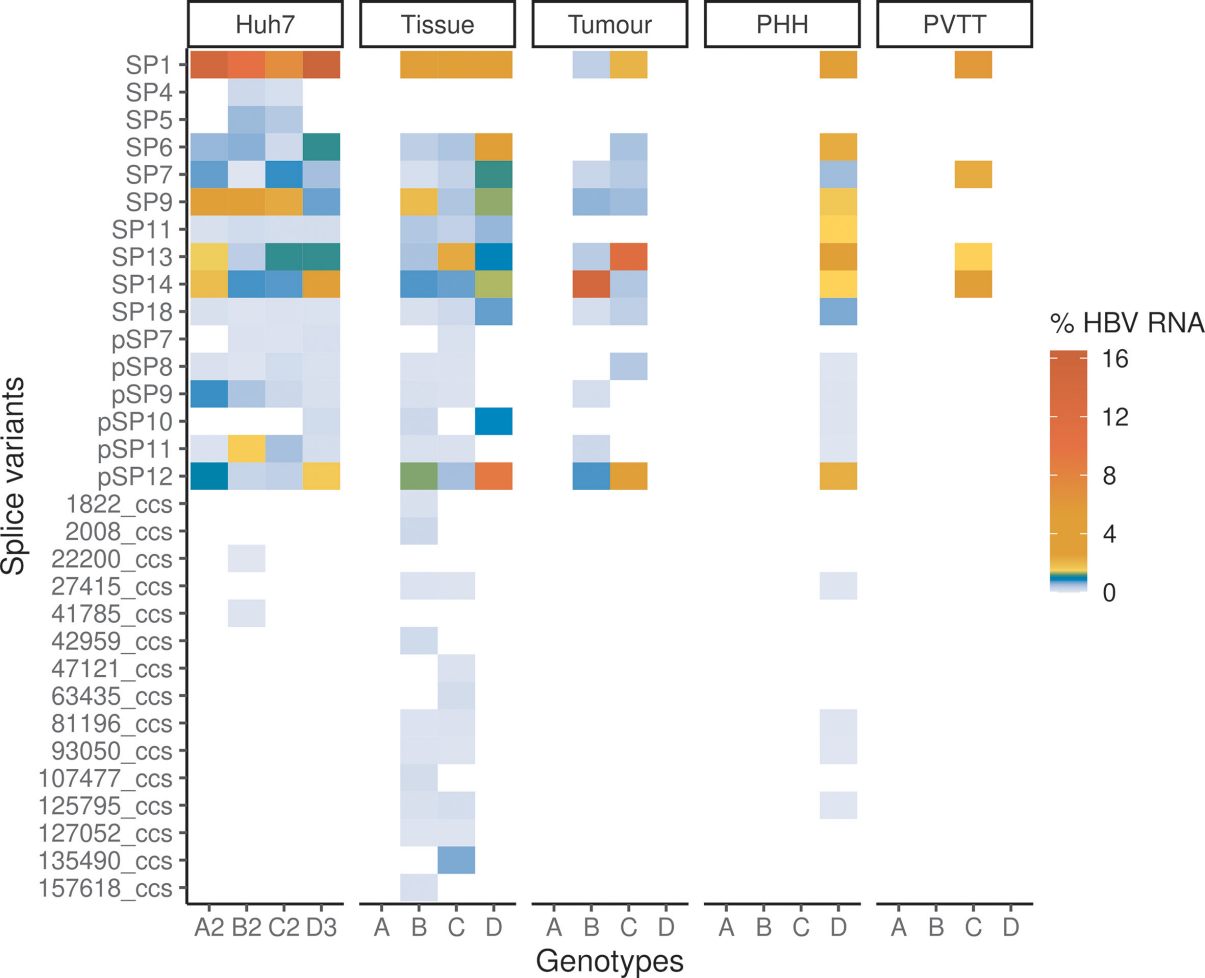
Expression profiles of HBV splice variants in HBV-transfected Huh7 cells, HBV-infected PHHs and biopsy samples. The heatmaps show the mean percentages of HBV RNAs that were spliced. The RNA-seq libraries that had ≥5 splice variants are shown. The known (SP) and putative (pSP) splice variants were reproducibly detected across the independent biological replicates of HBV-transfected Huh7. Other splice variants are represented with PacBio CCS read names (see Methods). See also Figs S4–S6, Table S1.

Previously reported splice variants SP1, 4, 5, 6, 7, 9, 11, 13, 14 and 18 were detected in the HBV genotypes [30, 33, 41, 43, 55] (Figs 2–4, Table S5). Notably, these known splice variants were consistently detected in all four genotypes, except for SP4 and SP5. As expected, SP1 was the major spliced transcript detected, ranging from 7.2 to 17 % of the HBV transcriptomes, which is in agreement with previous findings [30, 32, 89]. SP9 was the second most abundant spliced transcript, ranging from 0.9 to 4.7 % of the HBV transcriptomes. We also detected high but variable levels of SP13 and SP14 across the genotypes, whereas SP6 was the next most abundant.

### Condition- and genotype-specific expression profiles of splice variants in human liver and infected primary cells

To explore the biological relevance of these splice variants identified in a transfection model, we analysed 501 publicly available RNA-seq libraries (Table S1). These came from a diverse range of studies from HBV-positive liver biopsy samples, and HBV-infected PHHs, and different cell lines (HepaRG and HepG2-NTCP).

Significantly, most of the splice variants detected in Huh7 cells were detected in the biopsy samples and PHHs (Figs 4, S5 and S6, Table S1). Our analysis showed that the novel (pSP12) and known (SP1, SP6, SP9, SP13 and SP14) splice variants were also expressed at high levels in these clinical samples and PHHs.

Furthermore, liver tumour and portal vein tumour thrombosis (PVTT) (portal vein invasion at advanced-stage cancer) samples expressed lower levels of HBV RNAs than non-neoplastic tissue samples (Fig. S7, Table S6). This has been observed in independent studies [90–92], suggesting that HBV replication is less active in tumours. Strikingly, tumour samples expressed SP13 at significantly higher proportions of HBV RNAs than other samples except for PVTT (Figs 4 and S5, Wilcoxon rank sum tests, Bonferroni–Holm adjusted *P* value <0.05). Notably, SP13 encodes a P-S FP that has been shown to inhibit HBV replication [54].

Interestingly, we observed some genotype-specific expression profiles of splice variants across the different systems (HBV-transfected Huh7 cells, non-neoplastic liver tissue samples and HBV-infected PHHs; Figs 4 and S5). In particular, HBV genotype D expressed pSP12 and SP6 at significantly higher proportions of HBV RNAs than other three genotypes (Wilcoxon rank sum tests, Bonferroni–Holm adjusted *P* value <0.05).

Overall, we obtained higher numbers of uniquely mapped reads to HBV in Huh7 cells, followed by PHHs, non-neoplastic tissue, tumour and PVTT samples (Fig. S7, Table S6). The HBV read counts per library were also more reproducible in HBV-transfected Huh7 cells. In contrast, we detected low numbers of HBV reads in HBV-infected HepaRG and HepG2-NTCP cells. However, their median library sizes were a quarter or a third larger than other libraries (Fig. S7, Table S6).

### Sequencing depth has little effect on splice variant detection

To investigate how sequencing depth affects splice variant detection, we analysed the correlation between the number of unique RNA-seq reads mapped to HBV and library size. Strikingly, we observed weak correlations between the number of uniquely mapped reads (and spliced reads) to HBV and library size (Fig. S7, Kendall’s Tau coefficients of 0.20 and 0.13, *P* values of 5.6×10^−11^ and 1.7×10^−5^, respectively). Moreover, HBV spliced reads between biopsy samples could differ by an order of magnitude (Table S6). These results show that splice variant detection differs with genotypes and/or experimental conditions rather than only sequencing depth.

We observed that sequence alignment using a matched HBV genotype is critical in splice variant detection (Fig. S8). A total of 103 of 449 RNA-seq libraries have no uniquely mapped reads to HBV genotype A2 with 11 476 reads missing on average. By mapping to the corresponding HBV genotypes, we were able to detect splice variants in 267 of 501 publicly available RNA-seq libraries. Therefore, mapping RNA-seq reads to the HBV reference sequence may not be an appropriate approach and may partly explain the lack of prior reporting. However, most of the reports describing these 501 libraries did not look for splice variants and had other foci.

Taken together, the above findings suggest that Huh7 and PHH systems are more suitable for studying splice variants than clinical samples as: (i) HBV transcription and splicing were more reproducible in Huh7 and PHH systems than other biological materials and systems (Figs S5 and S7, Table S6), (ii) HBV RNAs in clinical samples could be very complex due to the presence of HBV quasispecies (in particular with deletions; Fig. S6), and (iii) the expression of HBV–human chimeric genes as a result of HBV integration [9, 91 ].

### Deletions at the X reading frame or BCP detected in HBV-positive biopsy samples

Deletions and splicing in the X reading frame have been reported previously [31, 45, 46, 93–95]. A careful examination of the RNA variants detected revealed three distinct deletions at the X reading frame or BCP (Fig. S6, Table S7). The deletion at positions 1757–1777 (20 bases) was the most common, which was detected in 6 of 41 HCC patients from two continents [7, 9]. In contrast, the 1719–1740 and 1749–1770 deletions (21 bases) were each detected in only one HCC patient. Two of these variants 1719–1740 and 1757–1777 co-locate with canonical splice sites (GU-AG), although they could be attributed to DNA mutations.

Interestingly, two of these deletions (1757–1777 and 1749–1770) were previously identified in the DNA of HBV quasispecies (Table S7) [93–95]. No matches were found for the 1719–1740 deletion, this in-frame deletion is novel. These findings suggest that aberrant splicing may be an avenue for the generation of quasispecies, in which the aberrantly spliced pgRNAs may be packaged and reversed transcribed. Moreover, deletions at X/BCP may contribute to the development of HCC [23, 96].

### Sequence variations surrounding the HBV splice sites affect splicing efficiency

We next investigated whether the sequence contexts of splice sites would be predicted to contribute to the different types and abundance of splice variants observed across genotypes. MaxEntScan scoring of the HBV splice sites showed that sequence variation was predicted to affect the strength of the splice sites of the different HBV genotypes (Fig. 3, Table S8).

In general, splice sites with weak sequence contexts (negative MaxEntScan scores) were less likely to be used for splicing and vice versa. For example, the splice donor site at position 2087 in genotypes A2 and D3 had poor sequence contexts and spliced reads associated with this site were not detected (see ① in Fig. 3, Table S8). In contrast, the same donor position in genotypes B2 and C3 had strong sequence contexts and were supported by over 100 spliced reads. This indicates that the splicing efficiencies of the HBV RNAs are strongly influenced by the HBV sequence variants surrounding the splice sites. Indeed, SP5 was not detected in the genotypes A2, D3 and a patient, who was a chronic carrier of HBV genotype D.

However, we also observed a discordance between splice site sequence contexts and splice read counts. For example, all the genotypes had similar scores at the splice acceptor position 1385, but the splice read counts were markedly different (see ② in Fig. 3, Table S8). In particular, the most frequently used 5′ splice site that was used for SP1 and SP9 had a negative MaxEntScan score (see ③ or position 2447 in Fig. 3, Table S8).

Taken together, these results suggest that the splicing of this splice junction may be controlled by other *cis* regulatory elements, such as the HBV post-transcriptional regulatory RNA element (PRE) [19]. Indeed, deleting a PRE component called the splicing regulatory element-1 (SRE-1) was previously found to inhibit pgRNA splicing and the production of SP1 [89]. Regulation of alternative splicing may play a crucial role in viral–host interactions [97].

### HBV encoded more alternative 3′ splice sites than 5′ splice sites

**Fig. 5.**
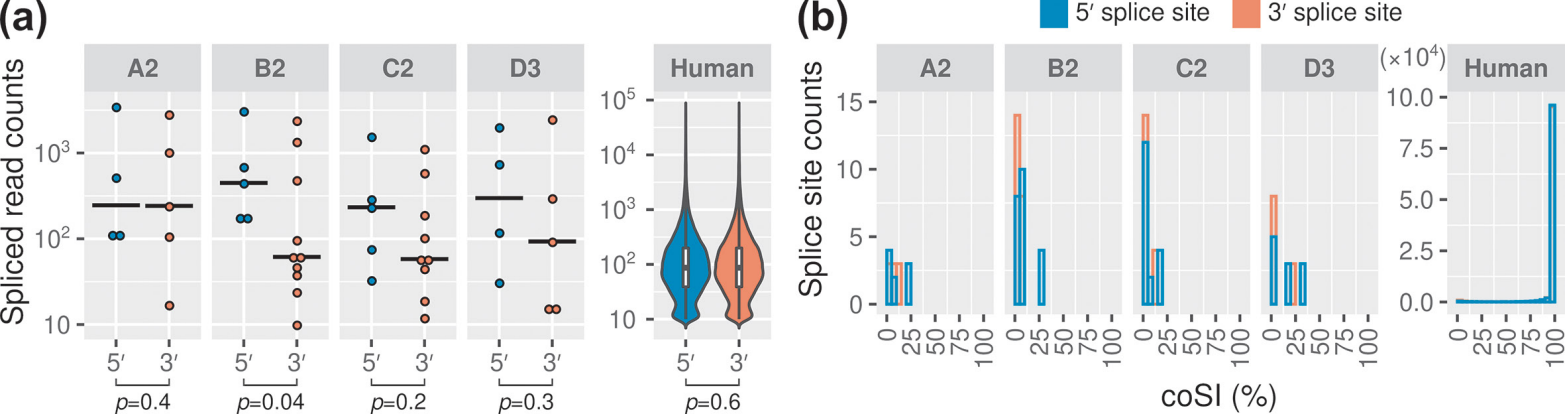
HBV 5′ splice sites are more likely to be spliced than 3′ splice sites. (a) More spliced reads were mapped across the 5′ splice sites of HBV than 3′ splice sites. Similar results were obtained from Welch two-sample *t*-test (one-sided) and permutation test (e.g. *P* values of 0.04 and 0.06 were obtained for genotype B2, respectively). Solid black lines indicate median values. (b) Completeness of splicing at the 5′ and 3′ splice sites. Only the splice sites supported by ≥10 reads were included for comparison.

A closer examination of the HBV splicing profiles revealed that HBV encoded more alternative 3′ splice sites than 5′ splice sites (Figs 3, S4 and S6, Table S8). Indeed, a trend was observed for more spliced reads mapped across the 5′ splice sites than 3′ splice sites, which reached statistical significance for HBV genotype B2 (Fig. 5). In contrast, host RNAs had balanced numbers of 5′ and 3′ splice sites (53 009 and 52 998, respectively), as well as the supported read counts (Fig. 5, median read counts of 87 for both the 5′ and 3′ splice sites).

To quantify the rates of splicing in HBV versus host cell RNAs, we scored the 5′ and 3′ splice sites using coSI. The 5′ splice sites of HBV showed higher coSI scores than 3′ splice sites (9 % versus 5 % on average; see also Fig. 5). In contrast, splicing was 87 and 86 % completed at the host 5′ and 3′ splice sites, respectively. These results showed that the 5′ splice sites of HBV tend to be more frequently spliced than 3′ splice sites (e.g. see ② in Fig. 3), but were much less efficiently utilized than host splice sites.

**Fig. 6.**
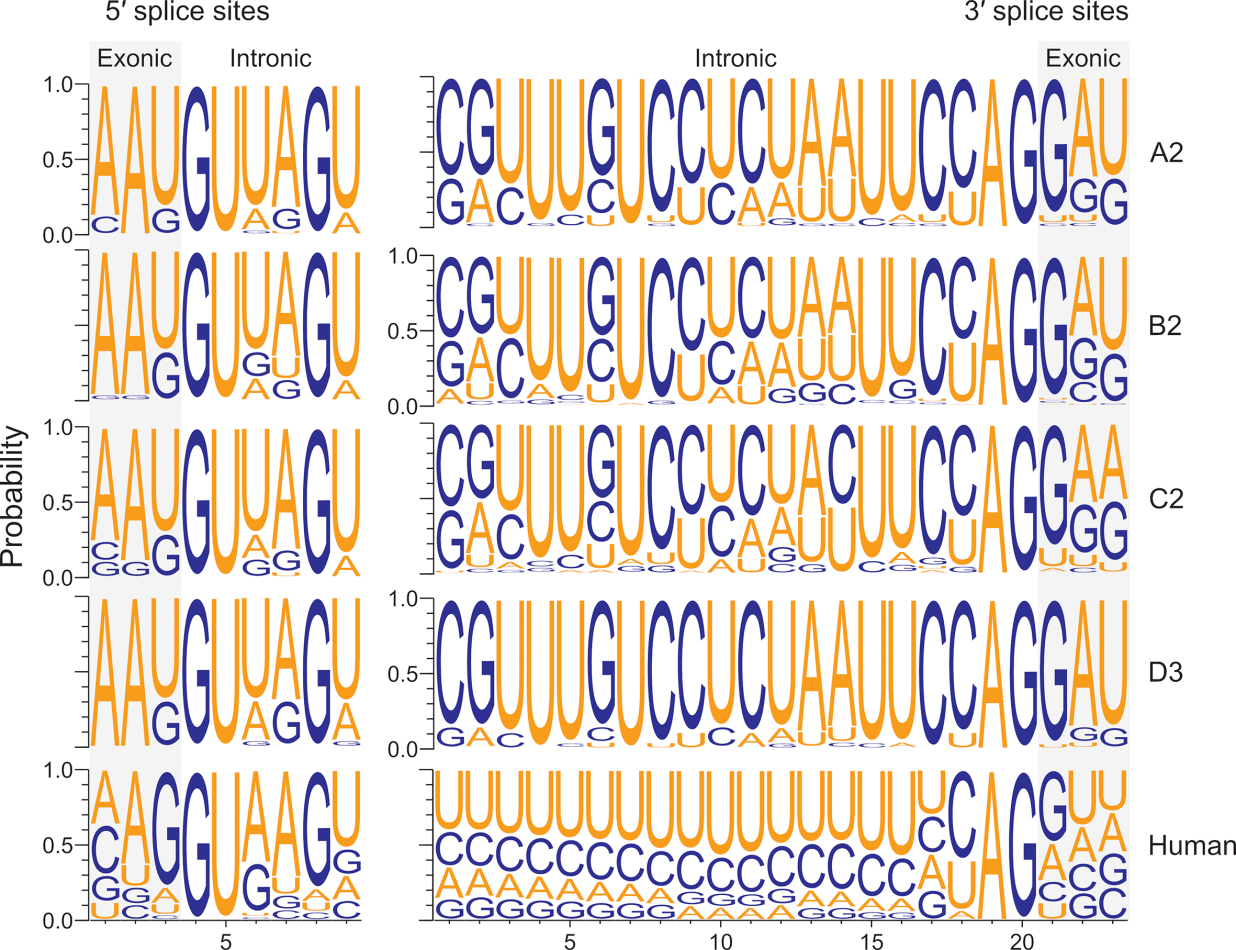
Most frequently used splice sites differed between HBV and the host. The nucleotide frequencies surrounding the splice sites are represented by the spliced reads. Exon boundaries are shaded in grey. Only the splice sites supported by ≥10 reads were included.

To identify the key differences between the HBV and human genomic splice sites, we compared their splice site contexts using the frequencies of the uniquely mapped, spliced reads to estimate the most frequently used splice sites. This approach showed that the nucleotide frequency distributions of human splice sites were similar to previous studies (Fig. 6) [98]. Notably, the most frequently used splice sites differed between the virus and host, e.g. −1 positions of the splice donor sites (Fig. 6, left panel, U versus G shaded in grey). The differences between the HBV genotypes were marginal, as the spliced reads were predominantly mapped to SP1 and SP9 (Figs 2b and 3).

### HBV replication had little effect on host gene expression in a transfection model

**Fig. 7.**
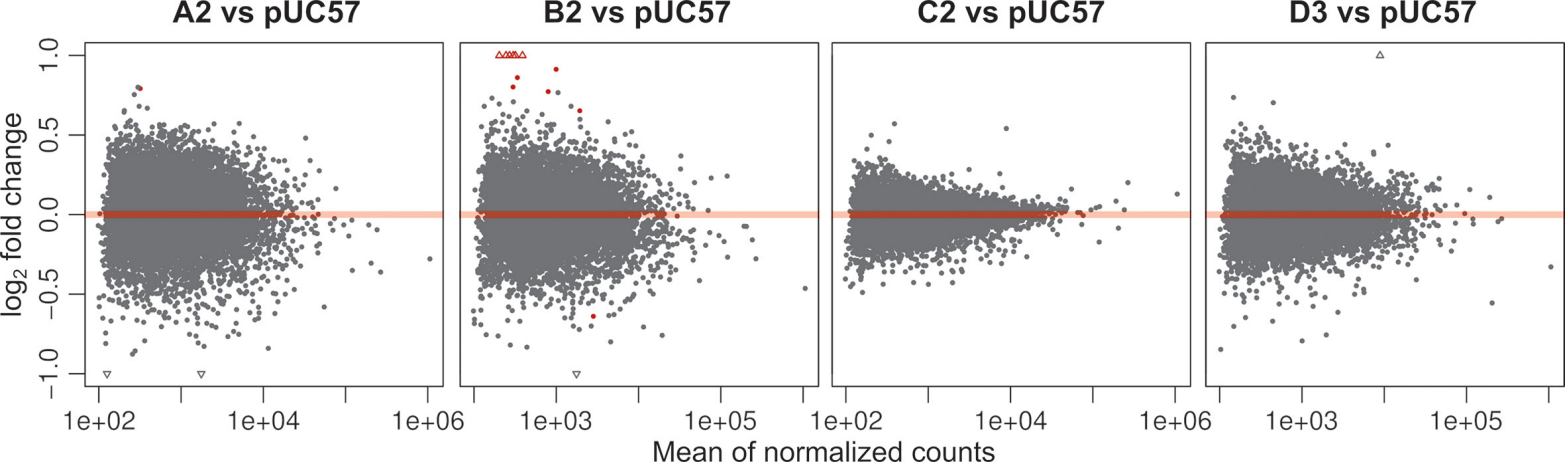
MA plots [log ratio versus mean expression (log scale)] show differential gene expression between the HBV-treated and control samples. Normalized counts indicate the counts divided by the normalization factors (as computed using the deseq2 default function). Red points denote the FDR-adjusted *P* value of <0.05. Unfilled triangles denote the genes that have undergone twofold changes in expression. See also Table S9.

**Fig. 8.**
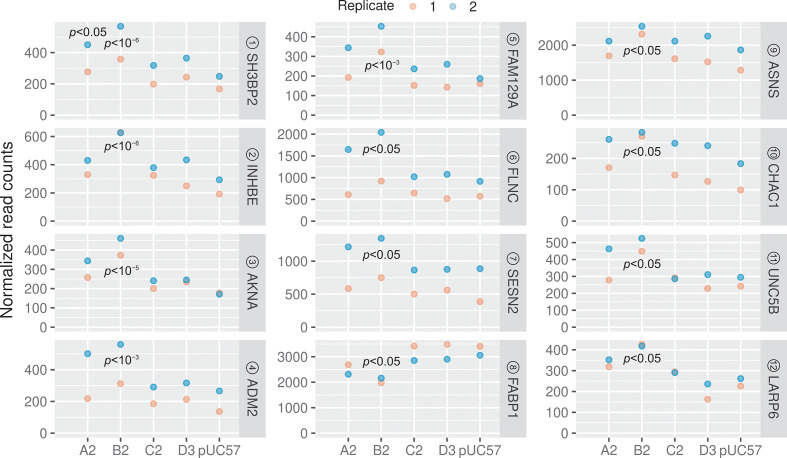
Significantly dysregulated genes in the HBV-treated cells. A total of 12 genes were differentially expressed in the B2-treated sample. Circled numbers denote the ranking based on FDR-adjusted *P* values. See also Tables S9 and S10.

HBV genomes that were transfected into cells could potentially have significant effects on cellular gene expression, even after only 24 h. To understand the impact of HBV replication on the host, we carried out differential gene expression analysis using deseq2 [82]. We found that only 1 and 12 genes were significantly differentially expressed in A2 and B2 treated samples, respectively, compared to the empty plasmid control [Figs 7 (red points) and 8, Table S9, FDR-adjusted *P* value <0.05]. Interestingly, both the A2 and B2 genotypes also showed relatively higher levels of HBV transcriptomes than the C2 and D3 genotypes (Fig S3, Tables S4 and S5). The accumulation of HBV transcripts may induce a stress response as the stress-related genes INHBE, FAM129A, SESN2, ASNS and CHAC1 were all upregulated (Fig. 8; see also Table S10 for functional annotation). Indeed, previous studies have also shown that HBV infection could lead to endoplasmic reticulum stress [99–101], including upregulation of INHBE [8, 102]. Interestingly, three significantly upregulated genes (ADM2, AKNA and SH3BP2) were previously shown to correlate with the Ishak fibrosis stage [103]. In particular, ADM2, a gene that is involved in ADORA2B-mediated production of anti-inflammatory cytokines, was also previously found to be differentially expressed in liver tumours [3].

### Concluding remarks

Our study has shed light on the complexity of splicing in four major HBV genotypes in cell lines and patient samples. We identified a number of novel splice variants, as well as previously identified variants, by mapping RNA-seq reads to specific HBV genotypes. Although previous studies have shown that HBV splice variants can be encapsidated, it is expected that the resulting virus particles are defective [1, 37, 39, 40, 51]. While this may apply to the splice variants with a disrupted P reading frame, the deletions or aberrant splicing that we have detected in X/BCP may produce viable quasispecies – their full-length genomic DNAs have been previously sequenced [93–95]. These deletions may be contributing to the development and/or recurrence of HCC [49, 56, 57].

We acknowledge that this study is limited to one member of each of the genotypes, and needs to be expanded to include additional HBV genotypes and subgenotypes. Nonetheless, this study demonstrates that HBV has a large capacity for alternative splicing, likely controlled by *cis*-acting elements such as the PRE [19], which results in high-levels of SP1 and SP9 mRNAs, despite the suboptimal context of the 5′ splice site. The role of the SP9 variant in particular needs to be further explored. With up to a quarter of all HBV mRNAs being of spliced origin, the importance of these molecules in the HBV ‘life cycle’ and pathogenesis requires further investigation.

## Supplementary Data

Supplementary material 1

Supplementary material 2
