# Supplementary material for: Quantitative analysis of the splice variants expressed by the major hepatitis B virus genotypes
Source: Microb Genom. 2021 Jan 13;7(1):mgen000492. doi: 10.1099/mgen.0.000492 (PMC8115900; doi:10.1099/mgen.0.000492)
Supplement: Supplementary material 1 [file mgen-7-492-s001.pdf]

SUPPLEMENTARY MATERIALS

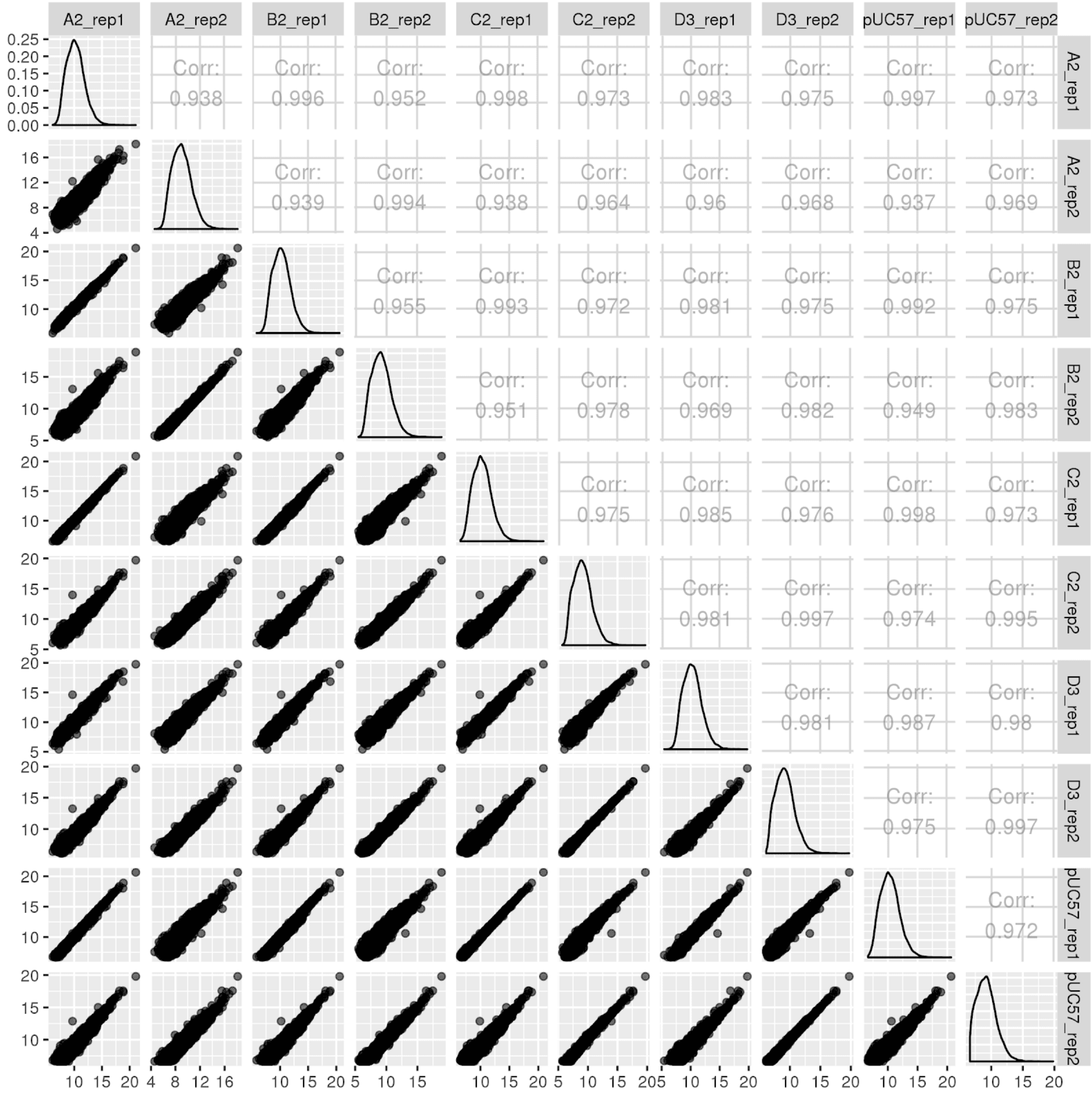

Fig S1. Correlation matrix plot of the RNA-seq read count data. Spearman correlations of >0.9 between biological replicates indicate good reproducibility. However, the Spearman correlations between the biological replicates are weaker than those within the same batch (i.e. rep1 versus rep2 < rep1 versus rep1, or rep2 versus rep2). This suggests that batch effects are present. rep, biological replicate.

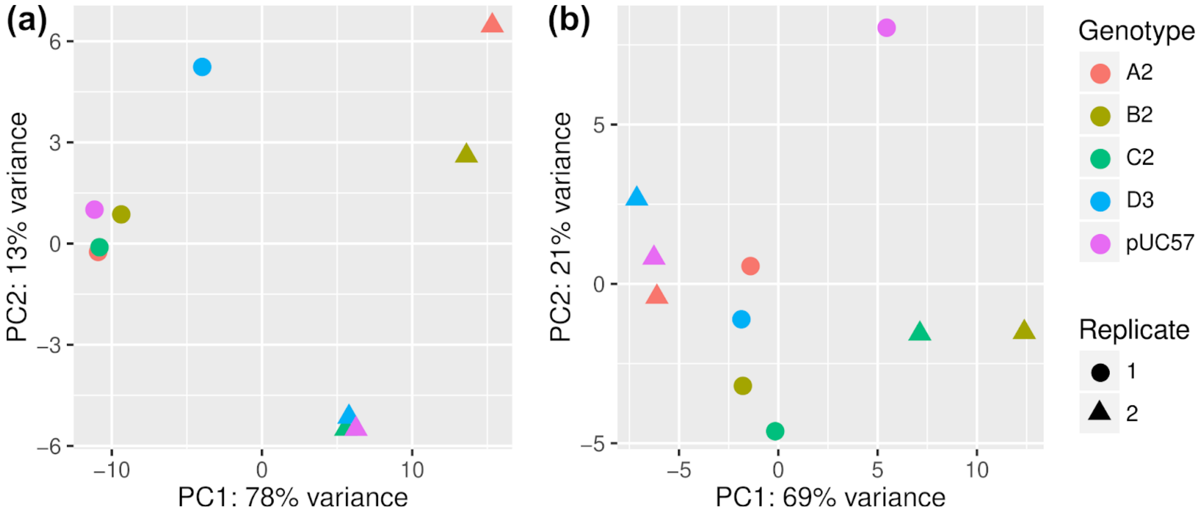

Fig S2. Batch effects in the RNA-seq experiments. (a) PCA plot shows that the replicates tend to be clustered by batch rather than genotype. (b) The read count data are distributed more randomly after a batch effect correction. PCA, principal component analysis.

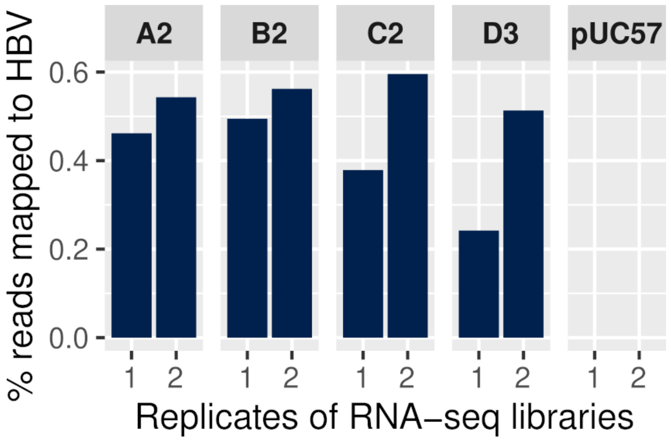

Fig S3. Proportions of the RNA-seq reads that were mapped to the HBV genomes by biological replicates. pUC57 empty plasmid was included as a control. See also Table S4 and the mapping statistics for the human genome in Table S2 and S3.

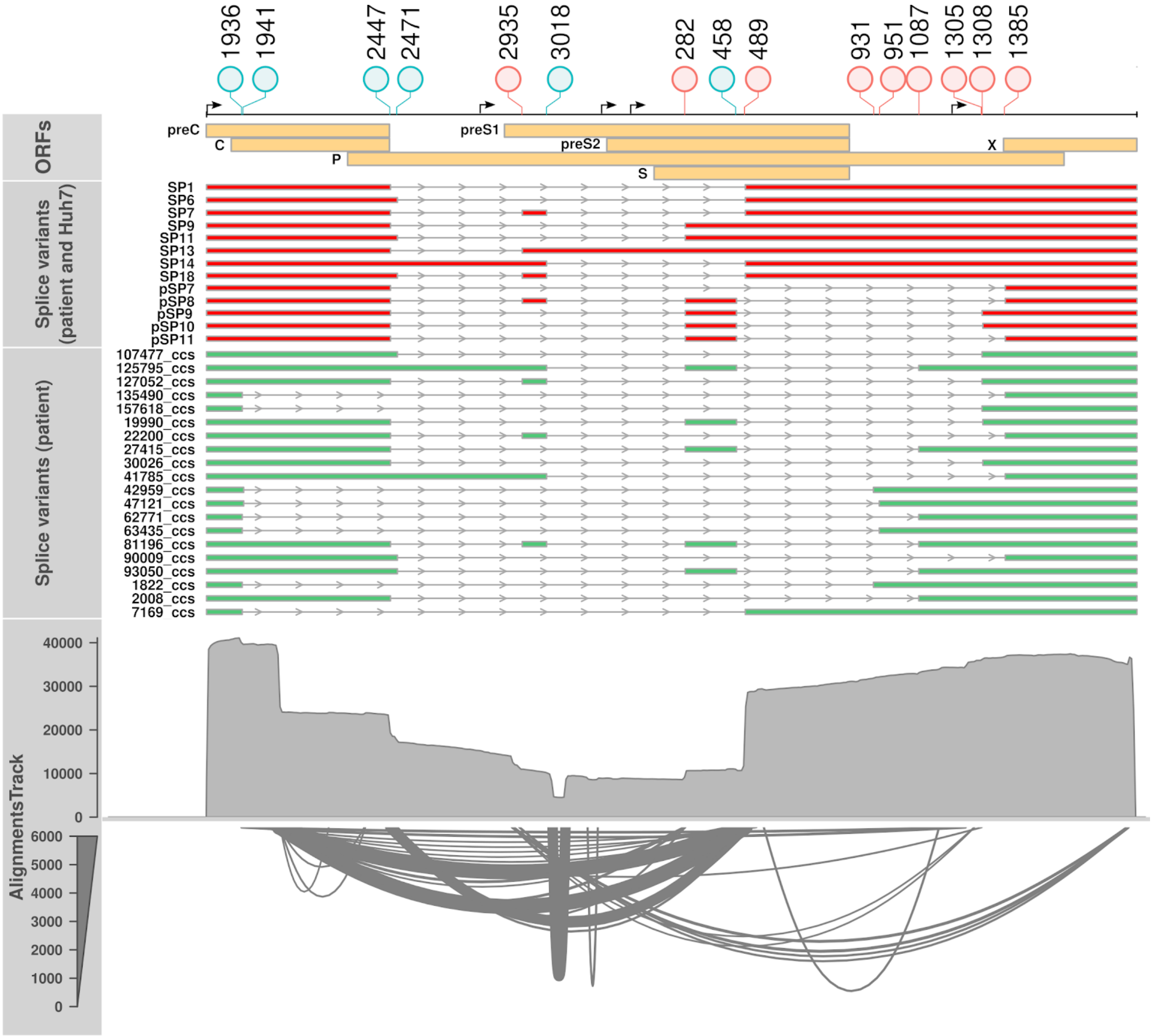

Fig S4. Known and novel HBV splice variants detected in a patient with chronic HBV infection. The HBV genomic DNAs were previously amplified from a patient's liver explant and blood samples and subjected to PacBio RS sequencing [1]. The publicly available CCS reads were used to identify splice variants (Methods). The splice variants labeled as pSP (putative splice variants) were detected in both the patient and HBV-transfected Huh7. They were also reproducibly detected across the independent biological replicates of this transfection experiment. The splice variants detected in the patient only are represented with PacBio CCS read names. Read coverage and splice graphs are shown in the bottom panel. CCS, Circular Consensus Sequencing.

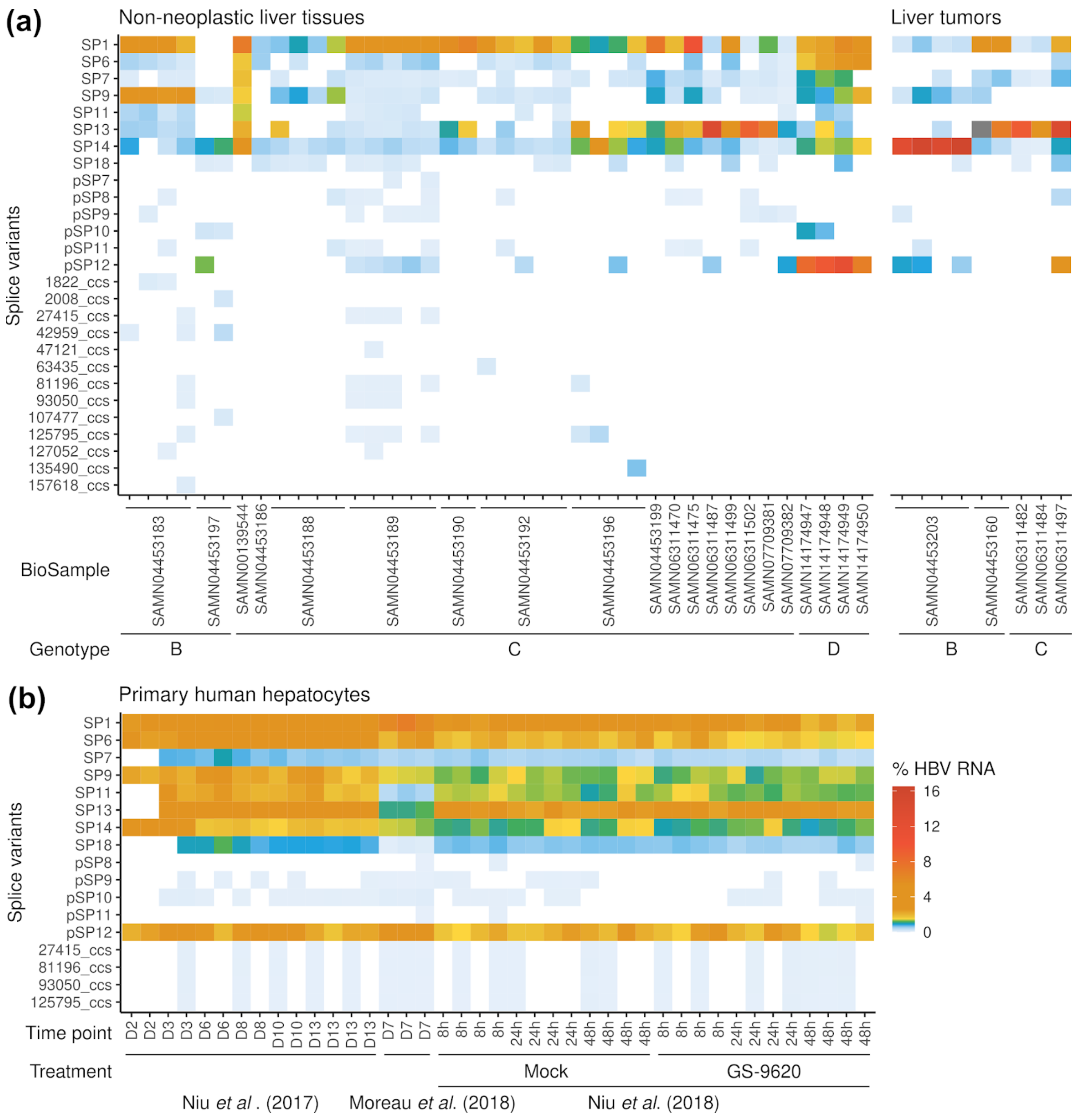

Fig S5. Expression profiles of HBV splice variants. The heatmaps show the percentages of splice variants in HBV RNAs in (a) HBV positive liver biopsy samples and (b) HBV-infected primary human hepatocytes. The RNA-seq libraries that had ≥5 splice variants are shown. SP and pSP denote the known and putative spliced pgRNAs, respectively. Other splice variants are represented with PacBio CCS read names (Methods). Mock indicates no GS-9620 treatment. CCS, Circular Consensus Sequencing.

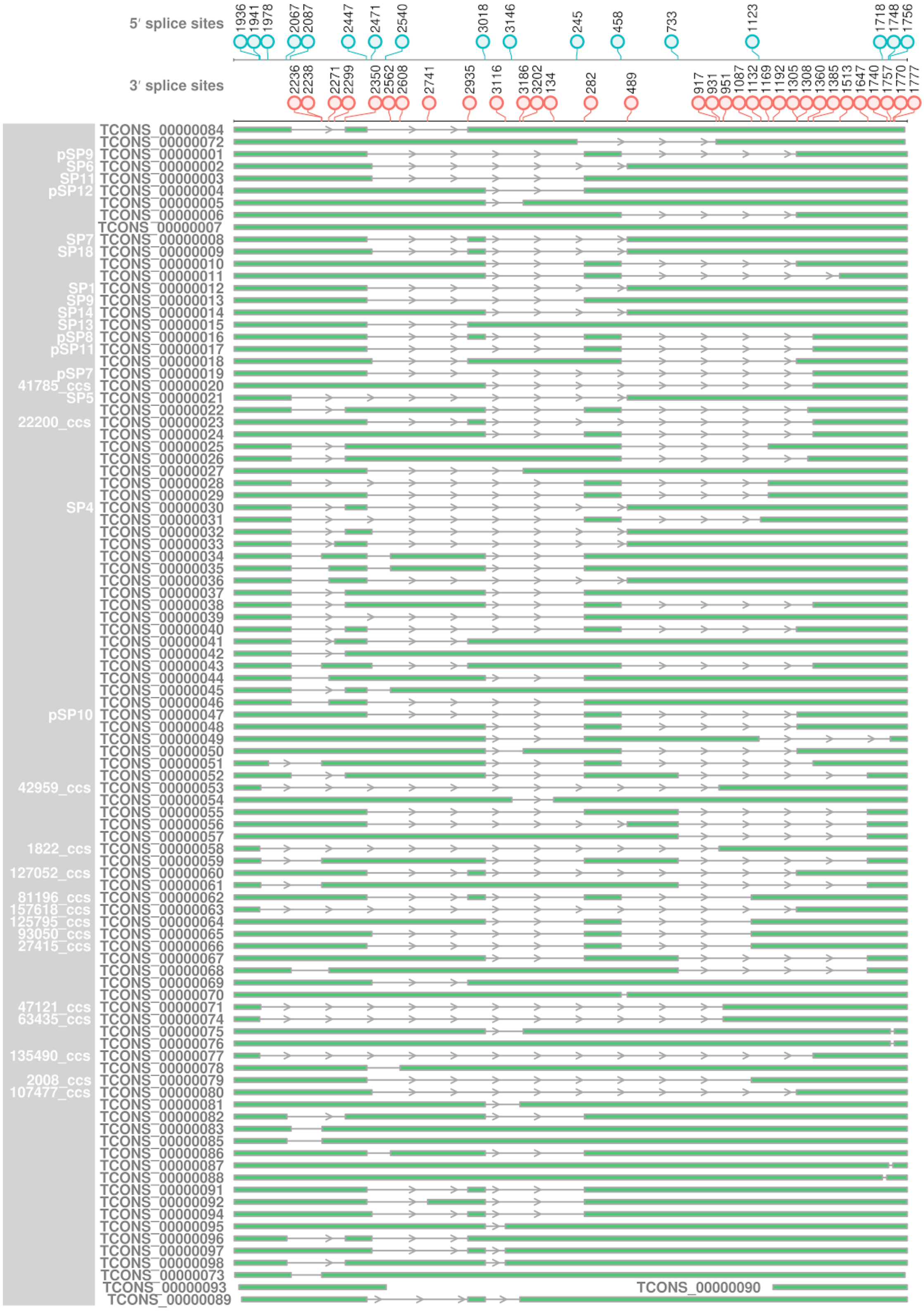

Fig S6. HBV transcripts assembled and detected in 513 RNA-seq libraries. This analysis included the 12 RNA-seq libraries of HBV-transfected Huh7 cells and 501 publicly available RNA-seq libraries of HBV positive biopsy samples of human liver tumors and non-neoplastic liver tissues, and PVTT (129, 182, and 92 libraries, respectively), as well as HBV-infected PHH (83 libraries) and human cultured cells HepaRG (4 libraries) and HepG2-NTCP (11 libraries). See also Table S1. PVTT, portal vein tumor thrombosis; PHH, primary human hepatocytes.

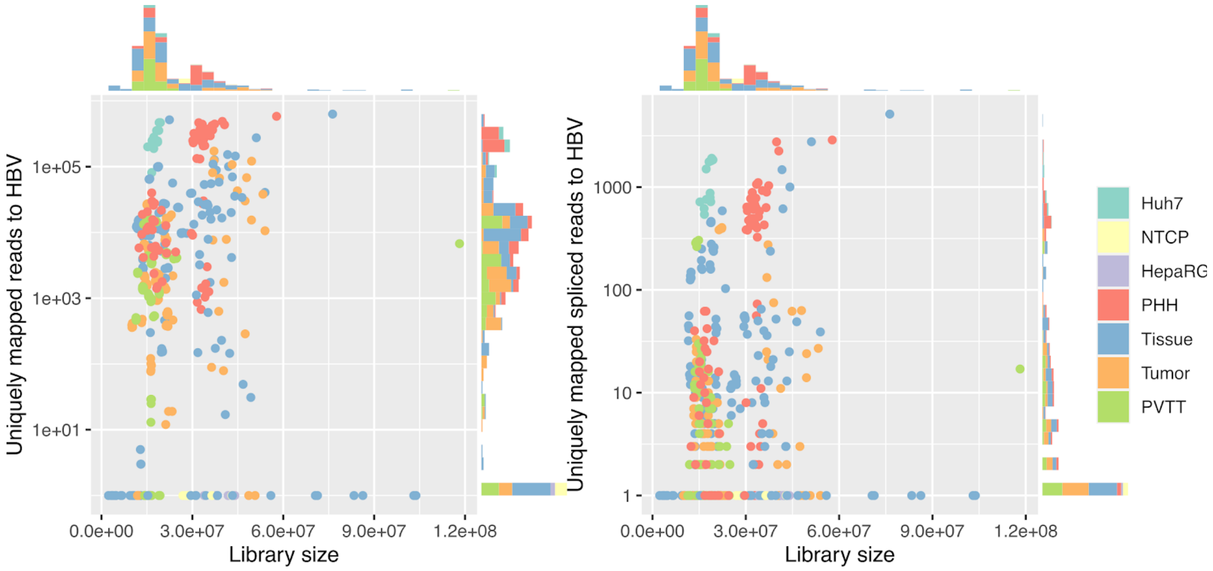

Fig S7. Weak correlation between the number of uniquely mapped reads to HBV and RNA-seq library size (Kendall's Tau = 0.20, *p*-value = 5.6×10^{-11}, left panel). The number of uniquely mapped, spliced reads to HBV also shows a weak correlation with library size (Kendall's Tau = 0.13, *p*-value = 1.7×10^{-5}, right panel). A pseudocount of 1 was added to read counts so that the y-axis can be shown in log-scale. A total of 78 and 185 of 501 public RNA-seq libraries lack uniquely mapped reads and spliced reads mapped to HBV, respectively. See also Table S6.

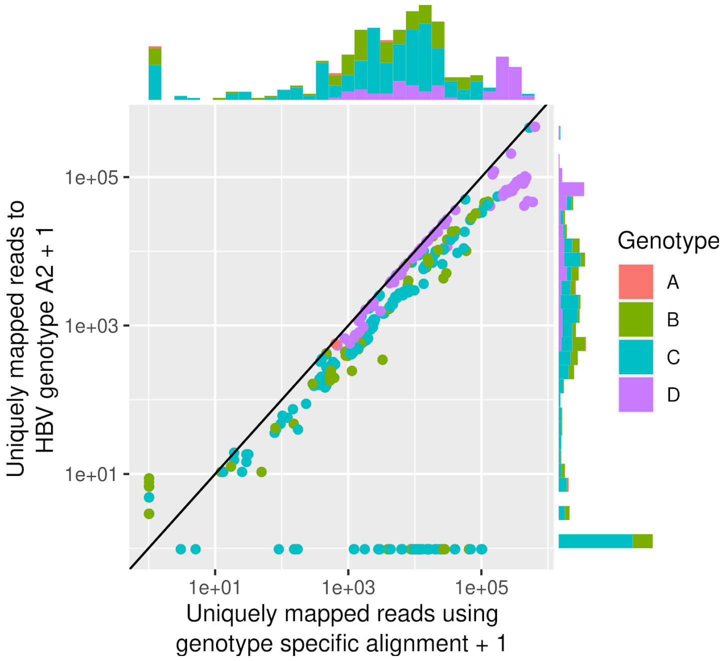

Fig S8. Suboptimal alignment of RNA-seq reads to incorrect HBV genotype. A pseudocount of 1 was added to the read counts so that the axes can be shown in log-scale. A total 103 of 449 RNA-seq libraries show no uniquely mapped reads to HBV genotype A2 with 11,476 reads missing on average. The black line indicates a perfect concordance between two variables.

Table S2. Alignment statistics of the RNA-seq libraries.

Library of HBV genotype (replicate)	Raw read pairs	Adapter-trimmed read pairs	Mapped reads (percent of adapter-trimmed read pairs)			
			Multiple loci	Too many loci	Unique	Unique, deduplicated
A2 (1)	37106899	37103731	3556481 (9.6%)	40667 (0.1%)	32136169 (86.6%)	21400178 (57.7%)
A2 (2)	15251776	15250842	1378840 (9.0%)	7998 (0.1%)	13205270 (86.6%)	11029753 (72.3%)
B2 (1)	38476667	38473340	3975647 (10.4%)	18182 (0.1%)	32973128 (85.7%)	22622573 (58.8%)
B2 (2)	17284712	17284008	1618680 (9.4%)	9607 (0.1%)	15051431 (87.1%)	12537297 (72.5%)
C2 (1)	37060457	37057565	3377149 (9.1%)	45873 (0.1%)	32538555 (87.8%)	22283264 (60.1%)
C2 (2)	16697692	16697028	1332564 (8.0%)	6783 (0.0%)	14762063 (88.4%)	11989291 (71.8%)
D3 (1)	35319988	35318556	2982199 (8.4%)	16857 (0.1%)	31093385 (88.0%)	24806308 (70.2%)
D3 (2)	17264080	17262771	1349438 (7.8%)	6606 (0.0%)	15202972 (88.1%)	12561085 (72.8%)
pUC57 (1)	37940141	37938002	3501830 (9.2%)	102293 (0.3%)	33227680 (87.6%)	23087037 (60.9%)
pUC57 (2)	17612295	17611657	1398322 (7.9%)	7586 (0.0%)	15462152 (87.8%)	12717607 (72.2%)

Table S3. Distributions of the deduplicated, uniquely mapped reads.

Library of HBV genotype (replicate)	Bases passing filter (PF)	PF aligned bases	Genic and intergenic distributions (percent of PF aligned bases)				
			Ribosomal	CDS	UTR	Intronic	Intergenic
A2 (1)	5417738625	5220912565	6750 (0.0%)	2950223954 (56.5%)	1448305082 (27.7%)	481157926 (9.2%)	341219323 (6.5%)
A2 (2)	2776824250	2635883740	4000 (0.0%)	1518208411 (57.6%)	657872991 (25.0%)	278418845 (10.6%)	181379887 (6.9%)
B2 (1)	5727443750	5502837275	6000 (0.0%)	3031085315 (55.1%)	1534479844 (27.9%)	552574669 (10.0%)	384692017 (7.0%)
B2 (2)	3152006375	3014745663	5500 (0.0%)	1718370044 (57.0%)	781535562 (25.9%)	303592100 (10.1%)	211243051 (7.0%)
C2 (1)	5639239750	5433899292	6750 (0.0%)	3112898043 (57.3%)	1516955005 (27.9%)	472662154 (8.7%)	331377964 (6.1%)
C2 (2)	3008063000	2922664578	3250 (0.0%)	1645081961 (56.3%)	815409053 (27.9%)	255555983 (8.7%)	206614503 (7.1)
D3 (1)	6237043625	6013322317	6750 (0.0%)	3471071102 (57.7%)	1712251181 (28.5%)	492800183 (8.2%)	337193472 (5.6%)
D3 (2)	3152469875	3055313767	3250 (0.0%)	1698269138 (55.6%)	842967328 (27.6%)	293568716 (9.6%)	220505546 (7.2%)
pUC57 (1)	5846806250	5626092532	6750 (0.0%)	3244918197 (57.7%)	1566227765 (27.8%)	494963155 (8.8)	319977161 (5.7%)
pUC57 (2)	3191559500	3093717169	2500 (0.0%)	1719594807 (55.6%)	866469052 (28.0%)	297178541 (9.6%)	210472480 (6.8%)

Table S4. Abundance of the reads mapped to the HBV genotypes.

Library of HBV genotype (replicate)	Deduplicated, uniquely mapped reads	
	Human genome	HBV genome (%)
A2 (1)	43341909	200145 (0.5)
A2 (2)	22214594	120648 (0.5)
B2 (1)	45819550	226622 (0.5)
B2 (2)	25216051	141763 (0.6)
C2 (1)	45113918	170852 (0.4)
C2 (2)	24064504	143348 (0.6)
D3 (1)	49896349	120681 (0.2)
D3 (2)	25219759	129444 (0.5)
pUC57 (1)	46774450	0
pUC57 (2)	25532476	0

Table S5. Abundance of the HBV splice variants identified and quantified using StringTie[a].

Genotype	Splice variant	TPM	Remarks
A2	SP1	709.085	Known
B2	SP1	611	Known
C2	SP1	338.878	Known
D3	SP1	662.638	Known
A2	SP11	5.68785	Known
B2	SP11	11.8632	Known
C2	SP11	5.61062	Known
D3	SP11	4.01303	Known
A2	SP13	72.9895	Known
B2	SP13	19.0319	Known
C2	SP13	54.4802	Known
D3	SP13	47.3871	Known
A2	SP14	92.2638	Known
B2	SP14	41.0153	Known
C2	SP14	34.5716	Known
D3	SP14	195.439	Known
A2	SP18	2.70639	Known
B2	SP18	1.68298	Known
C2	SP18	1.67218	Known
D3	SP18	2.33704	Known
B2	SP4	7.87638	Known
B2	SP5	26.9434	Known
C2	SP5	16.8507	Known
A2	SP6	27.4146	Known
B2	SP6	40.7028	Known
C2	SP6	8.85518	Known
D3	SP6	47.4892	Known
A2	SP7	39.4037	Known

B2	SP7	1.5067	Known
C2	SP7	32.5956	Known
D3	SP7	15.5845	Known
A2	SP9	227.351	Known
B2	SP9	234.833	Known
C2	SP9	115.301	Known
D3	SP9	34.9994	Known
B2	pSP7	3.01437	CCS[b]
B2	pSP8	1.90303	CCS
C2	pSP8	5.78398	CCS
D3	pSP8	2.97602	CCS
A2	pSP9	34.3573	CCS
B2	pSP9	23.2681	CCS
C2	pSP9	8.09695	CCS
D3	pSP9	2.63816	CCS
D3	pSP10	4.47211	CCS
B2	pSP11	79.6986	CCS
C2	pSP11	12.7991	CCS
D3	pSP11	4.62553	CCS
A2	pSP12	57.0472	Novel
D3	pSP12	68.1513	Novel

[a]The total RNA levels of A2, B2, C2, and D3 are 4812, 5442, 4708, and 3972 TPM, respectively.

[b]HBV splice variants found in a patient with chronic HBV infection, who has undergone liver transplantation [1]. pSP1-7 were previously used as identifiers for predicted splice variants found in the liver explant and blood samples taken after transplantation during follow-up. We recovered more splice variants in this long-read dataset that were also identified in our short-read dataset and extended the list to pSP12. pSP13 and 14 were only identified in our dataset.

TPM, Transcripts Per kilobase Million mapped reads; CCS, PacBio Circular Consensus Sequencing reads.

Table S6. Summary of RNA-seq libraries of HBV studies. See also Fig S7.

Biological materials	Remarks	Number of libraries	Median library size	Total uniquely mapped reads to HBV		Uniquely mapped, spliced reads to HBV	
				Median	Mean (95% CI)	Median	Mean (95% CI)
PHH	HBV infection	83	31,564,144	29,824	155,167 (118,503 ± 191,831)	61	361 (242 ± 480)
Tissue	HBV+	182	19,972,807	9,380	25,922 (16,061 ± 35,783)	6	97 (31±164)
PVTT	HBV+	92	15,683,413	1,426	4,548 (3,314 ± 5,782)	1	17 (4 ± 29)
Tumor	HBV+	129	17,954,621	1,854	11,172 (6,677 ± 15,666)	1	21 (8 ± 34)
HepaRG	HBV infection	4	39,826,813	0	0	0	0
NTCP	HBV infection	11	28,996,844	0	0	0	0
Huh7 (this study)	HBV transfection	24	18,356,451	247,670	268,863 (189,567 ± 348,158)	832	1,152 (801 ± 1,504)

CI, confidence intervals.

Table S7. Deletions at the X reading frame or basal core promoter.

Positions (size, frame)	Potential splice site motif	Patient ID (genotype): BioSamples	Sample (spliced read counts[a])	Top BLAST hits
1719-1740 (21 bases, in-frame)	GU-AG	P131 (B): SAMN06311478	Tissue (41)	Novel.
1749-1770 (21 bases, in-frame)	GA-TT	P105 (C): SAMN06311475	Tissue (5)	GQ278142.1, KR014092.1, KJ173205.1 [2–4].
1757-1777 (20 bases, out-of-frame)	GU-AG	#20 (C): SAMN04453156, SAMN04453196, SAMN04453176	Tumor (1561), tissue (889), PVTT (1132)	EU916221.1, KY470924.1, KR013992.1 [2, 4, 5].
		#17 (C): SAMN04453153	Tumor (9)	
		#14 (C): SAMN04453170	PVTT (22)	
		P157 (C): SAMN06311484, SAMN06311470	Tumor (223), tissue (5)	

[a] BioSamples with at least 5 uniquely mapped, spliced reads to HBV were reported.

Table S8. Positions of the HBV 5′ and 3′ splice sites[a], MaxEntScan scores, and junction-spanning read counts[b]. See also Fig 3.

SS	A2			B2			C2			D3		
	Position	Score	Reads	Position	Score	Reads	Position	Score	Reads	Position	Score	Reads
3′	276	9.2	1024	282	9.2	1358	282	9.2	587	282	9.2	298
5′	452	9.8	107	458	9.8	691	458	9.8	76	458	9.8	31
3′	483	8.8	2833	489	8.8	2405	489	10.5	1121	489	8.8	2482
3′	1126	-2.9	NA	1132	4.6	10	1132	-3.8	NA	1132	-3.8	NA
3′	1163	-7.4	NA	1169	1.0	47	1169	1.4	NA	1169	1.7	NA
3′	1299	8.1	107	1305	8.1	97	1305	5.8	19	1308	4.6	17
3′	1354	-6.7	NA	1360	4.4	64	1360	4.4	NA	1360	-5.4	NA
3′	1379	3.9	NA	1385	4.1	484	1385	5.5	57	1385	3.9	14
5′	2081	-6.9	NA	2087	7.9	163	2087	7.9	233	2087	-8.8	NA
3′	2265	-10.0	NA	2271	-7.9	NA	2271	-9.7	103	2271	-9.5	NA
3′	2293	0.6	NA	2299	6.4	NA	2299	9.2	12	2299	0.7	NA
3′	2344	-3.0	NA	2350	-3.0	59	2350	-3.0	58	2350	-3.9	NA
5′	2447	-1.8	3479	2447	-1.8	3094	2447	-1.8	1560	2447	-1.8	2008
5′	2471	6.0	116	2471	6.0	190	2471	6.0	33	2471	5.9	119
3′	2562	-3.6	NA	2562	8.9	NA	2562	9.2	45	2562	-1.4	NA
3′	2935	4.3	242	2935	3.9	24	2935	5.4	190	2902	5.6	93
5′	3018	9.8	521	3018	6.0	448	3018	9.8	290	2985	9.5	746
3′	3202	-3.5	17	3202	2.8	38	3202	-3.5	NA	3169	2.9	NA

[a]Coordinates are relative to the unique *EcoR*I restriction site in genotype C2.
[b]Overhang counts as there are a total of 15 3′ splice sites sharing five 5′ splice sites. Only the splice junctions supported by >9 uniquely mapped reads with >24 overhang bases were included. NA, no data available or read counts <10; SS, splice site.

References

1. **Betz-Stablein BD, Töpfer A, Littlejohn M, Yuen L, Colledge D, *et al.*** Single-Molecule Sequencing Reveals Complex Genome Variation of Hepatitis B Virus during 15 Years of Chronic Infection following Liver Transplantation. *J Virol* 2016;90:7171–7183.

2. **Hao R, Xiang K, Peng Y, Hou J, Sun J, *et al.*** Naturally occurring deletion/insertion mutations within HBV whole genome sequences in HBeAg-positive chronic hepatitis B patients are correlated with baseline serum HBsAg and HBeAg levels and might predict a shorter interval to HBeAg loss and seroconversion during antiviral treatment. *Infect Genet Evol* 2015;33:261–268.

3. **Yin J, Zhang H, He Y, Xie J, Liu S, *et al.*** Distribution and hepatocellular carcinoma-related viral properties of hepatitis B virus genotypes in Mainland China: a community-based study. *Cancer Epidemiol Biomarkers Prev* 2010;19:777–786.

4. **Li Z, Xie Z, Ni H, Zhang Q, Lu W, *et al.*** Mother-to-child transmission of hepatitis B virus: evolution of hepatocellular carcinoma-related viral mutations in the post-immunization era. *J Clin Virol* 2014;61:47–54.

5. **Fang Y, Teng X, Xu W-Z, Li D, Zhao H-W, *et al.*** Molecular characterization and functional analysis of occult hepatitis B virus infection in Chinese patients infected with genotype C. *J Med Virol* 2009;81:826–835.
